# Nutritional prehabilitation in head and neck cancer patients (PreHead) – A randomized controlled trial study protocol

**DOI:** 10.1371/journal.pone.0346273

**Published:** 2026-04-15

**Authors:** D. Sijbrands, D. Gort-van Dijk, S.A.H.J. de Visscher, A. van der Hoorn, R.J.H.M. Steenbakkers, H. Jager-Wittenaar, G.H. de Bock, A.T. Zwart, G.B. Halmos, I. Wegner

**Affiliations:** 1 Department of Otorhinolaryngology and Head and Neck Surgery, University of Groningen, University Medical Center Groningen, Groningen, The Netherlands; 2 Department of Nutrition and Dietetics, University of Groningen, University Medical Center Groningen, Groningen, The Netherlands; 3 Department of Oral and Maxillofacial surgery and Head and Neck Surgery, University of Groningen, University Medical Center Groningen, Groningen, The Netherlands; 4 Department of Radiology, University of Groningen, University Medical Center Groningen, Groningen, The Netherlands; 5 Department of Radiation Oncology, University of Groningen, University Medical Center Groningen, Groningen, The Netherlands; 6 Research Group Healthy Ageing, Allied Health Care and Nursing, Hanze University of Applied Sciences, Groningen, The Netherlands; 7 Department of Gastroenterology and Hepatology, Dietetics, Radboud university medical center, Nijmegen, The Netherlands; 8 Department of Physiotherapy, Human Physiology and Anatomy, Faculty of Physical Education and Physiotherapy, Research Unit Experimental Anatomy, Vrije Universiteit Brussel, Brussels, Belgium; 9 Department of Epidemiology, University of Groningen, University Medical Center Groningen, Groningen, The Netherlands; PLOS: Public Library of Science, UNITED KINGDOM OF GREAT BRITAIN AND NORTHERN IRELAND

## Abstract

**Rationale:**

Up to 60% of patients with head and neck cancer are malnourished upon first presentation. Malnutrition has been associated with a higher risk of adverse events and decreased quality of life and survival. Patients with a high risk of malnutrition often receive pretreatment dietary treatment before surgery or (chemo)radiotherapy, i.e., nutritional prehabilitation. However, previous research suggests that patients with a low or medium risk of malnutrition may also benefit from nutritional prehabilitation.

**Objective:**

To investigate the effect of nutritional prehabilitation on adverse events, nutritional status, patient-reported quality of life, tumor recurrence, and (disease-specific and overall) survival. To evaluate the cost-effectiveness of nutritional prehabilitation compared with standard care.

**Study design:**

A single-center, non-blinded, randomized controlled trial.

**Study population:**

Patients with locoregionally advanced stage (III or IV) primary mucosal squamous cell carcinoma of the oral cavity, oropharynx, hypopharynx or larynx treated with curative intent and with a low or medium risk of malnutrition according to the Malnutrition Universal Screening tool.

**Sample size:**

Based on a power analysis, a total of 128 patients will be included.

**Intervention:**

The intervention arm will receive nutritional prehabilitation and the control arm will receive standard care (no nutritional prehabilitation).

**Main study parameters/endpoints:**

Adverse events (i.e., surgical complications and (chemo)radiotherapy toxicity). Complications will be measured within 30 days after surgery using the Clavien-Dindo classification. Toxicity will be evaluated using the Common Terminology Criteria for Adverse Events (CTCAE), 6 and 12 weeks after the start of (chemo)radiotherapy.

**Significance:**

It is hypothesized that nutritional prehabilitation, compared with no nutritional prehabilitation, will result in fewer (severe) adverse events, improvement of nutritional status, higher quality of life, equal risk of recurrence and better survival. It is hypothesized that the intervention will be cost-effective.

**Trial registration:**

The trial is registered in the Dutch Trial Register under registration number NL87676.042.24.

## Introduction

Head and neck cancer (HNC) is among the most frequent tumors in the world, with an estimated incidence of 835.000 new cases and 428.000 deaths in 2018 globally [1]. Patients with HNC are at risk of malnutrition due to the location of the tumor in the upper aero-gastrointestinal tract. Up to 60% of patients with HNC are malnourished upon first presentation [2–5]. Malnutrition has been associated with lower treatment tolerance, more postoperative complications, toxicity, lower quality of life and higher risk of mortality [6–8]. To identify patients in need of nutritional intervention, risk of malnutrition can be assessed using various screening tools, including the Malnutrition Universal Screening Tool (MUST) [9]. Dietary therapy, including counselling by a dietitian, is more often initiated in patients with a high risk of malnutrition, whereas patients with a low or medium risk of malnutrition do not routinely receive dietary therapy [10,11]. A recent study in patients with HNC has shown that postoperative complications occur more frequently in patients with a medium risk of malnutrition, but not in patients with a high risk of malnutrition [12]. This finding may be explained by the early and effective dietary interventions routinely provided to patients with a high risk of malnutrition. Patients with a low or medium risk of malnutrition may benefit from additional screening and subsequent nutritional prehabilitation.

To our knowledge, no previous study has prospectively evaluated personalized nutritional counselling as a form of prehabilitation in patients with HNC who are treated surgically or with (adjuvant) (chemo)radiotherapy in a randomized study design. Prehabilitation has been more extensively studied in other oncological disciplines. In patients with hepatobiliary, colorectal and upper gastrointestinal cancer treated with surgery, a meta-analysis found that duration of hospital stay was significantly shorter in patients participating in a prehabilitation program [13]. Although this meta-analysis did not find significant differences in complication or survival rates, other studies have noted a trend towards fewer complications [14].

Improving the identification and treatment of patients who need nutritional prehabilitation is still an unmet need in patients with HNC. It is hypothesized that nutritional prehabilitation will result in fewer (severe) treatment-related adverse events (i.e., intra- and postoperative complications, toxicity and mortality), a higher quality of life, an equal risk of recurrence and improved survival. It is hypothesized that the measure will be cost-effective.

### Objectives

The primary objective of the PreHead study (Prehabilitation in Head and neck cancer patients) is to investigate the effect of nutritional prehabilitation on adverse events (complication rates and (chemo)radiotherapy toxicity) in patients with locoregionally advanced HNC and with a low or medium risk of malnutrition.

Secondary objectives are to investigate the effect of nutritional prehabilitation on nutritional status, patient-reported quality of life, recurrence and (disease-specific and overall) survival and to evaluate the cost-effectiveness of nutritional prehabilitation compared with standard care.

## Materials and methods

### Study design and setting

A single-center, non-blinded, randomized controlled trial will be performed within the Department of Otorhinolaryngology – Head and Neck Surgery, Department of Oral and Maxillofacial Surgery – Head and Neck Surgery and the Department of Radiotherapy at the University Medical Center Groningen (UMCG). The UMCG is a tertiary referral head and neck oncology center in the Netherlands.

The Dutch HNC patient organization (Patiëntenvereniging Hoofd-Hals, PVHH) reviewed the complete study protocol including the patient information folder and has delivered input and feedback on the recruitment process, the secondary outcome measure ‘patient-reported outcome measures’, and ways of communicating study results to participants.

This protocol adhered to the SPIRIT 2025 guidelines [15].

### Eligibility criteria

Subjects will be drawn from our population of patients with HNC, using the in- and exclusion criteria mentioned below.

### Inclusion criteria

To be eligible to participate in this study, a subject must meet all of the following criteria:

- Primary mucosal squamous cell carcinoma;
- Located in the oral cavity, oropharynx, hypopharynx or larynx;
- Locoregionally advanced disease defined as stage III or IV according to the 8^th^ edition of the TNM Classification of Malignant Tumors [16]. For oropharyngeal cancer, HPV-negative TNM staging is used at the time of inclusion, regardless of the HPV status;
- Treated with curative intent;
- Low or medium risk of malnutrition based on the MUST;
- Pre-treatment CT scan performed up to 30 days before treatment;
- Age ≥18 years;
- Signed written informed consent.

### Exclusion criteria

A potential subject who meets any of the following criteria will be excluded from participation in this study:

- Previous surgical or radiotherapeutic treatment of the neck;
- Multiple simultaneous primary malignancies;
- Disability that could interfere with questionnaire fulfillment (e.g., not speaking Dutch).

### Sample size

A power analysis was performed to calculate the sample size needed. Intervention studies evaluating the effect of nutritional prehabilitation in patients with HNC have not been performed previously. Therefore, the numbers used for the power analysis are based on studies on the association between malnutrition and adverse events in patients with HNC. The rate of adverse events in the intervention arm is expected to be 15% based on the rates of adverse events in well-nourished study populations [8,17]. The rate of adverse events in the control arm is expected to be 40% based on reported rates of adverse events in malnourished patients [8] and reported odds ratios in studies on the association between sarcopenia, which is strongly associated with malnutrition, and adverse events [17,18]. To detect a clinically relevant difference of 25% between the intervention and control arm, with a two-sided alpha of 0.05 and a power of 80%, a minimum of 61 patients per arm are needed. With an expected drop-out of 5%, we will include a total of 128 patients. This analysis was performed using G*Power version 3.1.

### Randomization, blinding and treatment allocation

Included patients will be randomly assigned to one of two groups: nutritional prehabilitation or standard care. Randomization will be performed using the web-based randomization module provided by REDCap. Block randomization with a 1:1 allocation ratio will be applied. Patients will be stratified based on stage (III or IV) and tumor subsite (oral cavity, oropharynx, hypopharynx and larynx). The randomization chart, including block size, is established by an independent data manager before the start of the study. Consequently, treatment allocation sequence is concealed for patients, care providers and researchers (including outcome assessors). The outcome assessors in this study are patients (self-assessment using questionnaires) and care providers (Clavien-Dindo classification, Common Terminology Criteria for Adverse Events (CTCAE), recurrence and survival).

### Recruitment

Potentially eligible patients will be informed about the study by their treating physician, after which they will receive the patient information folder. Patients will be given one week to consider their decision. Signed written informed consent will be obtained prior to study participation.

### Study timeline

Participant recruitment commenced on May 15, 2025 and is expected to continue until May 2028. Data collection is expected to be completed by October 2030. The first study results are anticipated by the end of 2028.

### Intervention

According to current Dutch guidelines, dietary therapy, including counselling by a dietitian, is initiated in patients with a high risk of malnutrition, whereas patients with a low or medium risk do not routinely receive dietary therapy [10]. In this study, all patients with HNC are screened using the MUST at baseline. Patients with a high risk of malnutrition are treated by a dietitian as part of our current standard practice. Patients with a low or medium risk of malnutrition who meet the remaining eligibility criteria are eligible for inclusion. The nutritional prehabilitation intervention for this study is similar to the standard daily clinical care for patients with a high risk of malnutrition. Patients with a low or medium risk of malnutrition will be randomized to either the control or intervention group upon enrollment.

Nutritional prehabilitation includes nutrition counselling and education by a dietitian, oral nutritional supplements, and enteral and/or parenteral nutrition support, as appropriate for each individual patient and in accordance with the guidelines proposed by the World Health Organization (WHO), European Society for Clinical Nutrition and Metabolism (ESPEN), and the National Institute for Health and Care Excellence [11,19,20].

We aim for an energy intake of the resting energy expenditure (REE) plus 30–50% for physical activity level, illness and thermic effect of food. The equation of the Food and Agriculture Organization, WHO and United Nations University will be used to calculate the REE [19]. We aim for a protein intake of 1.2 to 1.5 g/kg/day in patients with a BMI of 20–25 kg/m^2^. For a BMI of less than 20 kg/m^2^, the protein requirement will be corrected to a BMI of 20 kg/m^2^ [21]. For a BMI of more than 25.0 kg/m^2^, Gallagher’s formula will be used to calculate protein requirement, according to local guidelines [22]. Vitamins, minerals and trace elements will be supplied in amounts equal to the recommended daily allowance and we will not be using high-dose micronutrients in the absence of specific deficiencies [20].

All patients randomized to the intervention group will receive dietary advice by a dietitian. Patients who are able to use oral intake are counseled to use an energy and protein enriched diet and will be offered oral nutritional supplements when necessary to meet estimated nutritional requirements. If oral intake is not possible or remains insufficient despite nutritional interventions (i.e., counselling and oral nutritional supplements), enteral nutritional support will be advised to the patient. Insufficient intake is defined as less than 50% of the requirement for more than one week or only 50–75% of the requirement for more than two weeks, and these are thus the indications for enteral nutritional support [20]. If enteral nutritional support is not sufficient or not feasible, we will advise parenteral nutrition support. Patients rarely need (par)enteral nutritional support in this prehabilitation stage.

The risk of developing refeeding problems will be established using the NICE guideline on nutrition support for adults [11]. In patients with a high risk of developing refeeding syndrome, nutrition support will be started at a maximum of 10 kcal/kg/day, increasing levels with 5 kcal/kg/day to meet or exceed full needs by four to seven days [11]. Vitamin B1 will be supplied in daily doses of at least 100 mg for at least five days as well as a balanced multivitamin and trace element supplement once daily for at least five days [23]. Potassium, phosphate and magnesium will be monitored and substituted, if necessary, by oral, enteral or parenteral route [11].

Dietetic consultations will be scheduled biweekly. Depending on the patients’ needs and preferences, the frequency of consultations may be increased, e.g., in case of weight loss or inability to use oral intake. These consultations may take place in-person at the outpatient department or through video or telephone calls. The first consultation takes on average 30 minutes, follow-up consultations on average 15 minutes. We expect that in 10–20% of included patients, dietary advice in the form of an energy and protein enriched diet will be sufficient and 80–90% of patients will be offered oral nutritional supplements to meet estimated nutritional requirements.

Nutritional prehabilitation will start as soon as patients are included in the study, which is within one and a half week after their first visit to the outpatient department, depending on the time needed to consider participation in the trial. The expected variation in duration of the intervention is on average three to four-and-a-half weeks. The prehabilitation intervention ends as soon as treatment for their HNC commences. Patients will still receive nutritional care as part of standard care when indicated (i.e., postoperatively and/or during (chemo)radiotherapy). Nutritional care given during treatment is the same as nutritional care that is outlined in this paragraph (i.e., given as a prehabilitation intervention). Patients undergoing (chemo)radiotherapy are all counseled by a dietitian during their treatment. Patients undergoing surgery will receive nutritional care if they are at a high risk of malnutrition (MUST score ≥2) or according to the guideline ‘perioperative nutritional management’ (i.e., inability to start oral intake within 48 hours after surgery or no more than 50% of the recommended daily energy intake on the fifth day postoperatively) [24].

Patients enrolled in the control arm of the study will receive standard care, which means they will not receive any nutritional counselling or nutritional intervention before treatment commences. They will receive nutritional counselling or intervention during (chemo)radiotherapy and postoperatively when indicated.

### Study parameters/endpoints

#### Primary outcomes.

The primary outcome will be adverse events (intra- and postoperative complications and (chemo)radiotherapy toxicity). Relevant complications include hemorrhage, infection and wound healing disorders including dehiscence and fistula. Complications will be measured within 30 days after surgery, using the Clavien-Dindo classification and evaluated during hospital stay and planned and unplanned outpatient visits [25]. Clavien-Dindo grade ≥2 complications will be deemed clinically relevant. Toxicity will be evaluated using the CTCAE, version 4.0, at 6 and 12 weeks after the start of (chemo)radiotherapy [26]. The following domains are scored within the CTCAE: xerostomia, taste, throat pain, oral pain, general pain, dysphagia, hoarseness and mucositis. Clinically relevant toxicity will be defined as CTCAE toxicity grade ≥ 3, irrespective of the mentioned domains.

Both complications and (chemo)radiotherapy toxicity are evaluated as part of our current standard practice.

#### Secondary outcomes.

The Patient-Generated Subjective Global Assessment Short Form (PG-SGA SF) [27,28], the Global Leadership Initiative on Malnutrition (GLIM) criteria [29], and bioelectrical impedance analysis (BIA) will be used to assess nutritional status at baseline, at the end of the dietetic intervention (or, for the control group, just before the treatment starts), and three months after surgery or (adjuvant) (chemo)radiotherapy. BIA measurements will be performed using the seca mBCA 525 device, a portable, multiple frequency medical body composition analyzer. Assessors will be trained by a dietitian.

Dietary intake will be recorded using a 24-hour recall or food diary to evaluate adherence and the effect of the intervention on protein and energy intake at baseline, at the end of the dietetic intervention (or just before the treatment starts), and three months after treatment. In the intervention group, a dietitian will assess the 24-hour recall at baseline and at the end of the intervention. Intervention group patients will be asked to fill in a food diary three months after treatment, while control group patients will be asked to fill in the food diary at all three measure moments.

Patient-reported quality of life will be assessed using the Eating Assessment Tool (EAT-10) [30], the European Organisation for Research and Treatment of Cancer (EORTC) Quality of Life Questionnaires Head and Neck 35-questions (EORTC H&N35) [31–33] and the Core 30-questions (EORTC C30) [33,34]. The quality of life questionnaires will be completed at baseline and at 3, 6, 12, 18 and 24 months after the end of treatment.

Recurrence and (disease-specific and overall) survival will be monitored for two years at intervals of three months. These intervals coincide with regular follow-up visits to the outpatient departments.

The EuroQol 5-Dimension 5-Level (EQ-5D-5L) is a patient-reported quality of life instrument and will be used for the economic evaluation of cost-utility and cost-effectiveness [35], together with the iMTA Medical Consumption Questionnaire (iMCQ) [36] and iMTA Productivity Cost Questionnaire (iPCQ) [37]. The EQ-5D-5L, iMCQ and iPCQ will be completed in the first year of follow-up at baseline and at 3, 6 and 12 months after last treatment.

#### Other study parameters.

Baseline characteristics include age, gender, smoking status, alcohol use, tumor site, tumor stage, HPV-status, (intended) type of treatment, height, weight (loss), Charlson Comorbidity Index, radiological sarcopenia, physical status using handgrip strength and frailty status. The presence of low skeletal muscle mass, also known as radiological sarcopenia, is defined as the skeletal muscle index (SMI) measured on a single CT slice at the level of third cervical vertebra using the method described by Swartz et al. [38]. The CT scans that are used for these measurements are part of our current standard diagnostic work-up. Evaluation of frailty status includes Timed Up and Go (TUG) [39], Six item Cognitive Impairment Test (6-CIT) [40], delirium risk, Patient Health Questionnaire-2 (PHQ-2) [41], recent falls, Instrumental Activities of Daily Living (Lawton-IADL) [42], MUST [9], Katz Index of Activities of Daily Living – 6 items (Katz-ADL-6) [43]. For patients undergoing nutritional prehabilitation, the duration of the nutritional prehabilitation intervention and number of dietetic consultations will be recorded. For surgical patients, length of hospital stay will be recorded.

### Participant timeline

**Fig 1:**
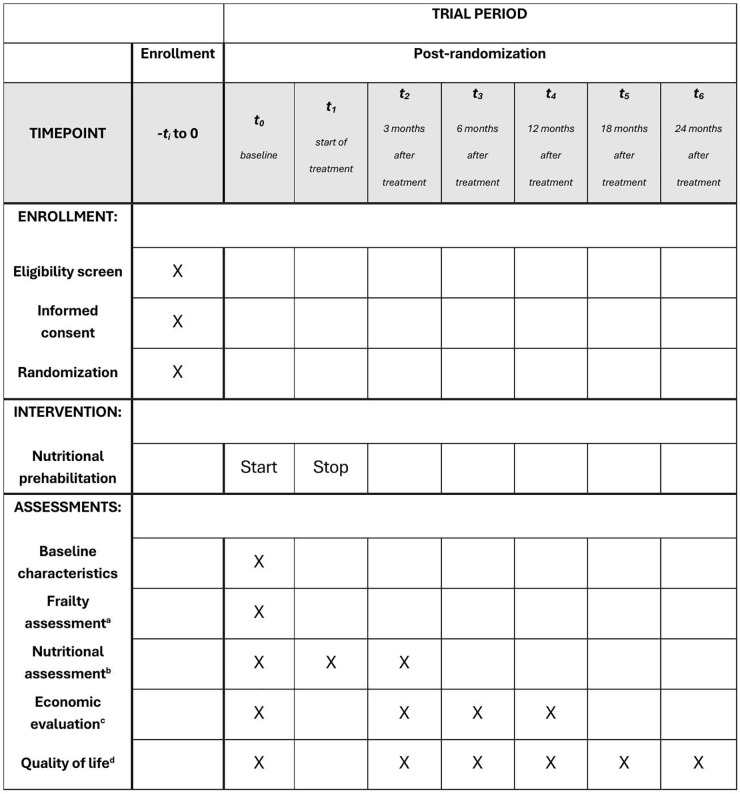
Participant timeline: SPIRIT schedule of enrollment, interventions, and assessments. ^a^Frailty assessment includes TUG, 6-CIT, delirium risk, PHQ-2, recent falls, iADL, MUST, Katz-ADL-6. ^b^Nutritional assessments include handgrip strength (only at t^0^), dietary intake using 24-hour recall or food diary, BIA, PG-SGA SF and GLIM-criteria. ^c^Economic evaluation questionnaires include EQ-5D-5L, iMCQ and iPCQ. ^d^Quality of life questionnaires include EAT-10, EORTC C30 and EORTC H&N35.

Fig 1 gives a summary of participant enrollment, interventions, and assessments (SPIRIT schedule).

### Harms and safety considerations

We expect that nutritional prehabilitation will lead to fewer (serious) complications and treatment-related side effects compared with no nutritional prehabilitation. We expect a higher quality of life, equal chance of recurrence, and better survival. There is extensive experience with the dietary measures. They are based on local, national and worldwide protocols and guidelines on nutritional support for patients with (head and neck) cancer [10,11,19,20]. All patients with a high risk of malnutrition based on the MUST receive dietary measures. Patients with high risk of malnutrition are more often frail [44], and more frequently experience swallowing problems than the study population, i.e., patients with a low or medium risk of malnutrition. The study population is a low risk patient group compared with the patient group already receiving the intervention. Oral nutritional supplements and enteral nutritional support used in this study comply with the FDA regulations regarding ingredients, labeling and manufacturing process.

Given the extensive experience with the intervention in a patient population at higher risk than the study population, and the small chance of adverse events that may cause slight damage at most, this study is considered a negligible risk study.

### Data collection and management

All data will be handled confidentially, and a patient identification code list will be used to link the data to the subject. All data will be collected in REDCap, a secured web application for managing online surveys and databases. Questionnaires will be sent through REDCap. In case patients prefer paper questionnaires, the questionnaire answers will be added to REDCap manually by one of the researchers.

The handling of personal data will comply with the EU General Data Protection Regulation and the Dutch Act on Implementation of the General Data Protection Regulation. Paper data will be stored for 15 years in a locked office at the UMCG, and digital data on a research drive especially made for research data storage.

### Statistical methods

Baseline characteristics, primary outcomes and secondary outcomes are quantitative and will be presented categorical and continuous. Categorical variables will be presented as absolute numbers and percentages of the total. Continuous variables will be tested for normality using the Kolmogorov-Smirnov test. Continuous variables will be presented as means and standard deviations for normally distributed data, and medians and interquartile ranges for data that is not normally distributed. Between-group mean (or median) differences, rate differences and rate ratios with 95% confidence intervals (or ranges) will be calculated.

Missing values will be handled using multiple imputation, assuming that there is only missingness at random, and all analyses will be performed on an intention-to-treat and per protocol basis.

Intercurrent events may affect the estimated treatment effect. An expected intercurrent event is non-adherence to the study intervention or the use of certain foods or medications outside the advice given by the dietitians. In the case of non-adherence to the study intervention or the use of certain foods or medications, a ‘treatment policy’ strategy will be used in which we accept that this intercurrent event is part of the intervention and we expect that non-adherence to the intervention also occurs in daily practice. Loss of follow-up because participants decide to stop the study is in our opinion not an intercurrent event but a ‘missing data’ problem. Patients who discontinue or deviate from the intervention protocol will be asked to complete follow up nonetheless.

SPSS version 28.0 (SPSS Inc., Chicago, IL, USA) will be used for all statistical analyses.

For analysis of between-group differences in the primary outcome, a logistic regression analysis will be performed. The endpoints complications and toxicity will be dichotomized. In case of unbalance between the intervention and the control group, adjusted logistic regression analyses will be performed. A *p*-value < 0.05 will be considered as statistically significant.

Subgroup analyses will be performed using the following variables:

- (Intended) type of treatment (surgery versus (chemo)therapy);
- Subsite (larynx versus hypopharynx versus oropharynx versus oral cavity);
- The presence of radiological sarcopenia or not (defined with the SMI in cm^2^/m^2^);
- PG-SGA SF;
- Duration of nutritional prehabilitation intervention;
- Number of dietetic consultations;
- Dietary intake using 24-hour recall.

These variables will be added to the regression analyses.

For further analyses of between-group differences within the secondary outcomes, logistic regression analyses will be performed for categorical outcomes, cox proportional hazard regression for time-dependent outcomes (i.e., recurrence and survival) and linear regression analyses for continuous variables. A *p*-value < 0.05 will be considered as statistically significant.

Furthermore, an economic evaluation will be performed to compare the incremental cost-effectiveness of nutritional prehabilitation as compared with standard care. A cost-utility analysis (CUA) and cost-effectiveness analysis (CEA) will be conducted from a societal perspective. The CUA will be performed based on EQ-5D-5L defined utilities over a time horizon of one year. The CEA will incorporate the highest measured grade of complications (Clavien-Dindo classification) at 30 days postoperatively or (chemo)radiotherapy toxicities (CTCAE) at 6 and 12 weeks after the start of (chemo)radiotherapy. Health care consumption will be measured on a patient level and registered on a case-report form and partly on questionnaires (iMCQ items). Productivity losses will be measured using the iPCQ. Costs will be calculated according to the Dutch guidelines for costing research in health economic evaluations, issued by the National Healthcare Institute [45]. There will be no discounting since the time horizon of the analyses does not exceed one year. Cost-effectiveness planes and acceptability curves will be plotted, and 95% confidence intervals will be based on bootstrapping.

### Nature and extent of the burden and risks associated with participation, benefit and group relatedness

Patients will be asked to undergo several measurements that are not part of our current standard practice. These measurements entail one handgrip strength measurement, three BIA-measurements and the following questionnaires: EAT-10, iMCQ and iPCQ at respectively six, four and four time points. Furthermore, the intervention itself will require up to three sessions with a dietitian, either during a regular visit at the outpatient department or through a telephone/video call. The time burden of these extra measurements and the intervention is 185 minutes on average.

There are negligible risks associated with the investigational treatment.

### Interim analysis

Not applicable.

### Ethics

The study will be conducted according to the principles of the Declaration of Helsinki (amended at the 64th WMA General Assembly, Fortaleza, Brazil, October 2013) and in accordance with the principles of ‘Good Clinical Practice’ and the Medical Research Involving Human Subjects Act (WMO). This study is approved by the Medical Ethical Committee (METc) of the UMCG and is registered in the Dutch Trial Register (CCMO registry NL87676.042.24, https://onderzoekmetmensen.nl/nl/trial/57366).

Protocol amendments will only be made after a favorable opinion by the accredited METc. In case the study is ended prematurely, the sponsor will notify the METc.

## Discussion

The PreHead study is the first randomized controlled trial to evaluate nutritional prehabilitation in patients with HNC. Previous research on prehabilitation in patients with HNC has primarily focused on swallowing interventions or supplementation (e.g., arginine), with limited evaluation of structured, personalized nutritional counselling tailored to individual energy and protein needs [46]. Studies that did examine the effect of nutritional counselling were all prospective cohort studies or case-control studies and only focused on patients undergoing (chemo)radiotherapy. For example, Paccagnella et al. retrospectively analyzed patients undergoing chemoradiotherapy who were referred for early nutritional intervention [47]. Although patients with a low risk of malnutrition received individualized nutritional counselling before and during radiotherapy, the number of pretreatment consultations and the exact start of the counselling were not specified, nor did they focus on a sufficient protein intake. A prospective study in patients with normal nutritional status undergoing chemoradiotherapy reported that early nutritional counselling improved survival and treatment tolerance [48]. All patients were advised to receive counselling aimed at adequate protein and caloric intake prior to and during treatment. However, the lack of randomization could have led to a selection bias. Outcome measures used within prehabilitation studies in HNC patients are diverse and no study measured outcomes focusing on the direct effects of prehabilitation, such as improvement in energy and protein intake.

However, our study has some limitations. First, we only evaluate the influence of nutrition. Prehabilitation programs in other specialties made a shift towards a more holistic approach of preparing patients for surgery. Multimodal prehabilitation programs cover multiple interventions targeting modifiable risk factors, such as nutritional care, improving aerobic fitness through an exercise program, and providing a psychological care program aiming to improve mental resilience [49]. Multimodal prehabilitation programs can work synergistically. A study regarding the feasibility of optimizing physical status during chemoradiotherapy in patients with HNC, involved a 10-week exercise program and showed limited feasibility (36% willingness to participate, 54% completion) [50], concluding that further research should focus on an adapted exercise program. A study covering the feasibility of exercise programs before a total laryngectomy is currently ongoing at the University Medical Center Utrecht, Utrecht, the Netherlands. A study examining the feasibility of a multimodal prehabilitation approach in HNC patients showed a participation rate of 60%, mainly caused by patients that are not willing to participate due to travel distance to training sessions [51]. Since both feasibility and effectiveness of exercise programs and dietary measures in a prehabilitation setting have not been sufficiently demonstrated in patients with HNC, it seemed to be better to investigate the effects of these individual interventions first. Combining the results of the PreHead study and available feasibility studies will lead to further development of tailored, multimodal prehabilitation programs for patients with HNC aiming at highest participation and lowest dropout rates.

A second limitation could be that informing patients about the potential benefits of nutritional prehabilitation may lead to behavioral changes on itself. Patients are likely to be more motivated to change their diet if they decide to participate. Patients randomized to the control group may seek nutritional education, counselling and support elsewhere or may consciously or subconsciously change their diet on their own. To evaluate changes in dietary intake in patients randomized to both the control and intervention groups, food intake is assessed (using a 24-hour recall or food diary) at baseline and two later time points to calculate differences in intake over time.

Third, dietary intake is assessed via a single 24-hour recall or food diary at three measurement moments to reduce participant burden. Although practical, this method may not fully capture habitual intake due to day-to-day variation.

### Dissemination policy

Manuscripts will be submitted to open-access, peer-reviewed journals, regardless of the results. There will be no restrictions towards publication. The results of this study will also be shared with patients and participants. At the termination of the study, PVHH will deliver input on ways of communicating study results to participants. A summary of the study findings rewritten into lay language will be distributed through the patient organization in their magazine and electronic newsletter.

## Supporting information

S1 File Completed SPIRIT 25 checklist. (DOCX)

S2 File Clinical Investigation Plan, version 3, September 4th, 2025. (PDF)
