# Supplementary material for: Nutritional prehabilitation in head and neck cancer patients (PreHead) – A randomized controlled trial study protocol
Source: PLoS One. 2026 Apr 15;21(4):e0346273. doi: 10.1371/journal.pone.0346273 (PMC13082693; doi:10.1371/journal.pone.0346273)
Supplement: S2 File — (PDF) [file pone.0346273.s002.pdf]

TEMPLATE RESEARCH PROTOCOL

(September 2018)

- May 2015: adaptation section 11.5: text in accordance to old and new Measure regarding Compulsory Insurance for Clinical Research in Humans
- Sept 2015: adaptation section 9.1, 9.2 and 12.5: text in accordance to WMO amendment on reporting SAE and temporary halt (section 10 of WMO)
- Oct 2015: adaptation section 4.4 – comment [CCMO15], 8.2 and 10.1 with respect to methodology/statistics
- Sept 2018: adaptation section 12.1 and comment [CCMO46] due to applicability GDPR as of May, 2018

Nutritional prehabilitation in head and neck cancer patients: a randomized controlled trial

**Protocol ID**	**NL87676.042.24**
**Short title**	**Nutritional prehabilitation in head and neck cancer**
**Version**	3
**Date**	4th September 2025
**Coordinating investigator/project leader**	**Dr. I. Wegner**<br>Department of Otorhinolaryngology – Head and Neck Surgery, UMCG
**Principal investigator(s) (in Dutch: hoofdonderzoeker/ uitvoerder)**	**Dr. I. Wegner**<br>Department of Otorhinolaryngology – Head and Neck Surgery, UMCG<br><br>**Dr. G.B. Halmos**<br>Department of Otorhinolaryngology – Head and Neck Surgery, UMCG
**Sponsor (in Dutch: verrichter/opdrachtgever)**	**University Medical Center Groningen**
**Subsidising party**	**KWF**
**Independent expert(s)**	**Dr. A.G.W. Korsten-Meijer**<br>Department of Otorhinolaryngology – Head and Neck Surgery, UMCG

PROTOCOL SIGNATURE SHEET

Name	Signature	Date
**Head of Department:**<br>**Prof. dr. H.A.M. Kerstjens**<br>Department of Otorhinolaryngology – Head and Neck Surgery, UMCG		**03-10-2025**
**Coordinating Investigator/Project leader/Principal Investigator:**<br>**Dr. I. Wegner**<br>Department of Otorhinolaryngology – Head and Neck Surgery, UMCG		**03-10-2025**

TABLE OF CONTENTS

LIST OF ABBREVIATIONS AND RELEVANT DEFINITIONS 6

SUMMARY 8

1. INTRODUCTION AND RATIONALE 9

2. OBJECTIVES 10

3. STUDY DESIGN 11

4. STUDY POPULATION 13

4.1 Population (base) 13

4.2 Inclusion criteria 13

4.3 Exclusion criteria 13

4.4 Sample size calculation 13

5. TREATMENT OF SUBJECTS 15

5.1 Treatment 15

5.2 Use of co-intervention (if applicable) 17

5.3 Escape medication (if applicable) 17

6. INVESTIGATIONAL PRODUCT 18

7. NON-INVESTIGATIONAL PRODUCT 18

8. METHODS 19

8.1 Study parameters/endpoints 19

8.1.1 Main study parameter/endpoint 19

8.1.2 Secondary study parameters/endpoints 19

8.1.3 Other study parameters 19

8.2 Randomisation, blinding and treatment allocation 21

8.3 Study procedures 21

Procedures that are part of standard clinical practice: 21

8.4 Withdrawal of individual subjects 23

8.4.1 Specific criteria for withdrawal (if applicable) 23

8.5 Replacement of individual subjects after withdrawal 23

8.6 Follow-up of subjects withdrawn from treatment 23

8.7 Premature termination of the study 23

9. SAFETY REPORTING **25**
9.1 Temporary halt for reasons of subject safety 25
9.2 AEs, SAEs and SUSARs 25
9.2.1 Adverse events (AEs) 25
9.2.2 Serious adverse events (SAEs) 25
9.2.3 Suspected unexpected serious adverse reactions (SUSARs) 26
9.3 Annual safety report 26
9.4 Follow-up of adverse events 26
9.5 Data Safety Monitoring Board (DSMB) / Safety Committee 27
10. STATISTICAL ANALYSIS **28**
10.1 Primary study parameter(s) 28
10.2 Secondary study parameter(s) 29
10.3 Interim analysis (if applicable) 30
11. ETHICAL CONSIDERATIONS **31**
11.1 Regulation statement 31
11.2 Recruitment and consent 31
11.3 Objection by minors or incapacitated subjects (if applicable) 31
11.4 Benefits and risks assessment, group relatedness 31
11.4.1 Risk assessment 31
11.5 Compensation for injury 32
11.6 Incentives (if applicable) 32
12. ADMINISTRATIVE ASPECTS, MONITORING AND PUBLICATION **33**
12.1 Handling and storage of data and documents 33
12.2 Monitoring and Quality Assurance 33
12.3 Amendments 33
12.4 Annual progress report 33
12.5 Temporary halt and (prematurely) end of study report 33
12.6 Public disclosure and publication policy 34
13. REFERENCES **35**

LIST OF ABBREVIATIONS AND RELEVANT DEFINITIONS

**AE**	Adverse Event
**AR**	Adverse Reaction
**BIA**	Bio-electrical impedance analysis
**CCMO**	Central Committee on Research Involving Human Subjects; in Dutch: Centrale Commissie Mensgebonden Onderzoek
**CEA**	Cost-Effectiveness Analysis
**CUA**	Cost-Utility Analysis
**CSMA**	Cross-sectional Skeletal Muscle Area
**CTCAE**	Common Terminology Criteria for Adverse Events
**DSMB**	Data Safety Monitoring Board
**ESPEN**	European Society for Clinical Nutrition and Metabolism
**EU**	European Union
**EWGSOP2**	European Working Group on Sarcopenia in Older People 2
**GDPR**	General Data Protection Regulation; in Dutch: Algemene Verordening Gegevensbescherming (AVG)
**GLIM**	Global Leadership Initiative on Malnutrition
**HU**	Hounsfield Unit
**LSMI**	Lumbar Skeletal Muscle Index
**METC**	Medical research ethics committee (MREC); in Dutch: medisch-ethische toetsingscommissie (METC)
**MUST**	Malnutrition Universal Screening Tool
**PG-SGA SF**	Patient Generated Subjective Global Assessment Short Form
**REE**	Resting Energy Expenditure
**(S)AE**	(Serious) Adverse Event
**Sponsor**	The sponsor is the party that commissions the organisation or performance of the research, for example a pharmaceutical company, academic hospital, scientific organisation or investigator. A party that provides funding for a study but does not commission it is not regarded as the sponsor, but referred to as a subsidising party.
**SUSAR**	Suspected Unexpected Serious Adverse Reaction
**TUG**	Timed Up and Go Test
**UMCG**	University Medical Center Groningen
**WHO**	World Health Organization

**WMO**	Medical Research Involving Human Subjects Act; in Dutch: Wet Medisch-wetenschappelijk Onderzoek met Mensen

SUMMARY

Rationale: up to 60% of patients with head and neck cancer are malnourished upon first presentation. Malnutrition has been associated with more adverse events and lower quality of life and survival. According to current Dutch guidelines, patient with high risk of malnutrition receive pretreatment dietary treatment. A recent study has shown that patients with low to medium risk of malnutrition, may benefit from additional screening and subsequent nutritional prehabilitation.

Objective: to investigate the effect of nutritional prehabilitation on adverse events in patients with locoregionally advanced head and neck cancer and with low to medium risk of malnutrition. To evaluate its effect on patient-reported quality of life outcome measures, recurrence and (disease-specific and overall) survival. To evaluate the cost-effectiveness of nutritional prehabilitation compared to standard care.

Study design: a single-center, non-blinded, randomized controlled trial.

Study population: patients with stage III and stage IV primary mucosal squamous cell carcinoma located in the oral cavity, oropharynx, hypopharynx or larynx treated with curative intent and with low or medium risk of malnutrition.

Intervention: one group will receive nutritional prehabilitation and one group will receive standard care (which means they will not receive nutritional prehabilitation).

Main study parameters/endpoints: adverse events (i.e., intra- and postoperative complications and (chemo)radiotherapy toxicity). Complications will be measured within 30 days after surgery using the Clavien-Dindo classification. Toxicity will be evaluated using the Common Terminology Criteria for Adverse Events, version 4.0, 6 weeks after the start of (chemo)radiotherapy.

Nature and extent of the burden and risks associated with participation, benefit and group relatedness:

Patients will be asked to undergo several measurements that are not part of our current standard practice. These measurements entail one grip strength measurement, three BIA-measurements and the following questionnaires: EAT-10, iMCQ and iPCQ at respectively six time points, four time points and four time points. Furthermore, the intervention itself will require three sessions with a dietitian, either during a regular visit at the outpatient department or through a telephone/video call. The time burden of these extra measurements and the intervention is 185 minutes on average.

There are negligible risks associated with the investigational treatment.

We expect that nutritional prehabilitation will lead to fewer (serious) complications and side effects compared to no nutritional prehabilitation. We expect a higher quality of life, equal chance of recurrence and better survival.

1. INTRODUCTION AND RATIONALE

Head and neck cancers are among the most frequent tumors in the world with an estimated 835.000 new cases and 428.000 deaths in 2018 globally [1]. Patients with head and neck cancer are eminently at risk for malnutrition due to the location of the tumor in the upper aero-gastrointestinal tract. Up to 60% of patients with head and neck cancer are malnourished upon first presentation [2-5]. Malnutrition has been associated with lower quality of life and treatment tolerance, and more postoperative complications, toxicity and mortality [6-8]. To identify patients in need of nutritional intervention, malnutrition risk is screened using the Malnutrition Universal Screening Tool (MUST) at the University Medical Center Groningen (UMCG). According to current Dutch guidelines, dietary therapy, including counseling by a dietitian, is started in patients with high risk of malnutrition, whereas patients with low to medium risk of malnutrition do not routinely receive dietary therapy [9]. A recent study in patients with head and neck cancer has shown that postoperative complications occur more frequently in patients with medium risk of malnutrition, but not in patients with high risk of malnutrition [10]. This is very likely to be explained by the effectiveness of dietary interventions in patients with high risk of malnutrition. Patients with low to medium risk of malnutrition, may benefit from additional screening and subsequent nutritional prehabilitation.

In patients with hepatobiliary, colorectal and upper gastrointestinal cancer treated with surgery, a recent meta-analysis found that duration of hospital stay was significantly shorter in patients participating in a prehabilitation program [11]. While no significant difference in complication rate or survival rate was found, other studies have noted a trend towards less complications [12]. Such intervention studies have not been performed in patients with head and neck cancer yet. **Efforts to better identify and treat patients with a need for nutritional prehabilitation thus minimizing treatment-related adverse events (i.e., intra- and postoperative complications, toxicity and mortality) and maximizing quality of life, represent an unmet need for patients with head and neck cancer.**

2. OBJECTIVES

Primary Objective: to investigate the effect of nutritional prehabilitation on adverse events (complication rates and (chemo)radiotherapy toxicity) in patients with locoregionally advanced head and neck cancer and with low to medium risk of malnutrition.

Secondary Objective(s):

To investigate the effect of nutritional prehabilitation on patient-reported quality of life outcome measures, recurrence and (disease-specific and overall) survival in patients with locally advanced head and neck cancer and with a low to medium risk of malnutrition;
To evaluate the cost-effectiveness of nutritional prehabilitation compared to standard care.

3. STUDY DESIGN

A single-center, non-blinded, randomized controlled trial will be performed. The study will be performed within the Department of Otorhinolaryngology – Head and Neck Surgery, Department of Oral and Maxillofacial Surgery and the Department of Radiotherapy at the University Medical Center Groningen.

Figure 1 visualizes the inclusion of patients. All head and neck cancer patients are screened using the MUST at baseline. Patients with high risk of malnutrition are treated by a dietitian as part of our current standard practice. Patients with low to medium risk of malnutrition are eligible for inclusion. As soon as they are enrolled, they will be randomized.

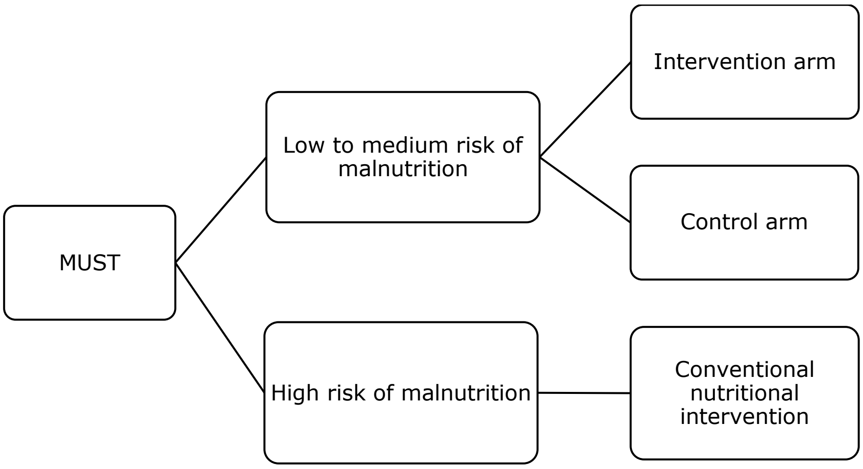

Figure 1. Study flow.
MUST = Malnutrition Universal Screening Tool.

Intervention
Included patients will be randomly assigned to one of two groups: nutritional prehabilitation or standard care. The nutritional prehabilitation intervention is not different from the treatment we offer patients with high risk of malnutrition based on the MUST in daily clinical practice. Nutritional prehabilitation will typically take around three to four-and-a-half weeks, given a pre-treatment time window in patients with head and neck cancer of four to five weeks from first referral to the outpatient clinic to the start of treatment. Further information on the intervention can be found under subheading 5.1 (treatment). Patients receiving standard care will not receive any nutritional counseling or nutritional intervention before treatment commences. Patients will be monitored for two years. Measurement moments will coincide

with regular follow-up visits to the outpatient departments. Further information on the outcomes to be measured can be found under subheading 8.1 (study parameters/endpoints).

4. STUDY POPULATION

4.1 Population (base)

Subjects will be drawn from our population of head and neck cancer patients, using the in- and exclusion criteria mentioned below. It is very likely we will be able to recruit the planned number of patients from the defined source population. We see on average 111 patients per year that are eligible for inclusion. Assuming a participation rate of 80%, we will be able to include the necessary 128 patients in approximately 17 months' time.

4.2 Inclusion criteria

In order to be eligible to participate in this study, a subject must meet all of the following criteria:

- Primary mucosal squamous cell carcinoma;
- Located in the oral cavity, oropharynx, hypopharynx or larynx;
- Locoregionally advanced disease defined as stage III and stage IV disease. For oropharynx malignancies the HPV-negative TNM-staging is used at the time of inclusion;
- Treated with curative intent;
- Low or medium risk of malnutrition based on the MUST;
- Pre-treatment CT scan performed up to 30 days before treatment;
- Age ≥18 years;
- Written informed consent.

4.3 Exclusion criteria

A potential subject who meets any of the following criteria will be excluded from participation in this study:

- Previous surgical or radiotherapeutic treatment of the neck;
- Multiple simultaneous primary malignancies;
- Disability that could interfere with questionnaire fulfillment.

4.4 Sample size calculation

We performed a power analysis to calculate the sample size needed. Intervention studies evaluating the effect of nutritional prehabilitation in patients with head and neck cancer have not been performed previously. Therefore, the numbers used for the power analysis are based on studies on the association between malnutrition/radiological sarcopenia and adverse events in patients with head and neck cancer. We expect the rate of adverse events in the intervention arm to be 15% based on the rates of adverse events in well-nourished/non-sarcopenic study populations [8, 13]. We expect the rate of adverse events

in the control arm to be 40% based on reported rates of adverse events in malnourished patients [8] and reported odds ratios in studies on the association between sarcopenia and adverse events [13, 14]. To detect a clinically relevant difference of 25% between the intervention and control arm, with a two-sided alpha of 0.05 and a power of 80%, a minimum of 61 patients per arm are needed. With an expected drop-out of 5%, we will include a total of 128 patients. This analysis was performed using G*Power version 3.1.

5. TREATMENT OF SUBJECTS

5.1 Treatment

Included patients will be randomly assigned to one of two groups: nutritional prehabilitation or standard care. The nutritional prehabilitation intervention is not different from the treatment we offer patients with high risk of malnutrition based on the MUST in daily clinical practice.

Nutritional prehabilitation includes nutrition counseling and education by a dietitian, oral nutritional supplements, and enteral and/or parenteral nutrition support, as appropriate for each individual patient and in accordance with the guidelines proposed by the World Health Organization (WHO), ESPEN and the National Institute for Health and Care Excellence [15-17]. An adequate intake will be supported that is based on the balance between the patient's total energy and protein needs, disease status, current intake, lifestyle and food preferences.

An energy and protein enriched diet will be advised. We aim for an energy intake of the resting energy expenditure (REE) plus 30 to 50% for physical activity level, illness and thermic effect of food. The equation of the Food and Agriculture Organization, WHO and United Nations University will be used to calculate the REE [15]. We aim for a protein intake of 1.2 to 1.5 g/kg/day in patients with a BMI of 20-25 kg/m^2. For a BMI of less than 20 kg/m^2, the protein requirement will be corrected to a BMI of 20 kg/m^2 [18]. For a BMI of more than 25.0 kg/m^2, Gallagher's formula will be used to calculate protein requirement, according to local guidelines [19]. Vitamins, minerals and trace elements will be supplied in amounts equal to the recommended daily allowance and we will not be using high-dose micronutrients in the absence of specific deficiencies 16].

Patients who are able to use oral intake, will receive dietary advice by a dietitian and will be offered oral nutritional supplements to meet estimated nutritional requirements. If oral intake is not possible or insufficient despite nutritional interventions (i.e., counselling and oral nutritional supplements), we will discuss enteral nutritional support with the patient. Insufficient intake is defined as less than 50% of the requirement for more than one week or only 50-75% of the requirement for more than two weeks, and these are thus the indications for enteral nutritional support [16]. If enteral nutritional support is not sufficient or not feasible, we will advise parenteral nutrition support.

If oral food intake has been decreased severely for a prolonged period; oral, enteral or parenteral nutrition will be increased slowly over several days and additional precautions

will be taken to prevent a refeeding syndrome. Patients who meet the following criteria are at high risk of developing refeeding problems according to the NICE guideline on nutrition support for adults [17]:

- One or more of the following criteria:
  - BMI <16 kg/m^2;
  - Unintentional weight loss >15% within the last three to six months;
  - Little or no nutritional intake for more than 10 days;
  - Low levels of potassium, phosphate or magnesium before feeding.
- Two or more of the following criteria:
  - BMI <18.5 kg/m^2;
  - Unintentional weight loss >10% within the last three to six months;
  - Little or no nutritional intake for more than five days;
  - A history of alcohol abuse or drugs including insulin, chemotherapy, antacids or diuretics.

In these patients, nutrition support will be started at a maximum of 10 kcal/kg/day, increasing levels with 5 kcal/kg/day to meet or exceed full needs by four to seven days [17]. Vitamin B1 will be supplied in daily doses of at least 100 mg for at least five days as well as a balanced multivitamin and trace element supplement once daily for at least five days [20]. Potassium, phosphate and magnesium will be monitored and substituted, if necessary, by oral, enteral or parenteral route [17].

Dietetic consultations will be scheduled biweekly. Depending on the patients' needs and preferences, the frequency of consultations may be increased, e.g., in case of weight loss or inability to use oral intake. These consultations may take place in-person at the outpatient department or through video or telephone calls. The first consultation takes on average 30 minutes, follow-up consultations on average 15 minutes. We expect that in 10-20% of included patients dietary advice in the form of an energy and protein enriched diet will be sufficient and 80-90% of patients will be offered oral nutritional supplements to meet estimated nutritional requirements. Patients rarely need enteral nutritional support in this prehabilitation stage.

Nutritional prehabilitation will start as soon as patients are included in the study, which is within one week after their first visit to the outpatient department, depending on the time needed to consider participation in the trial. The expected variation in duration of the intervention is three to four-and-a-half weeks. The **prehabilitation** intervention ends as

soon as treatment for their head and neck cancer commences. Patients will still be receiving nutritional care as is standard care (i.e., postoperatively and/or during (chemo)radiotherapy). Nutritional care given during treatment is the same as nutritional care that is outlined in this paragraph (i.e., given as a prehabilitation intervention). Patients undergoing (chemo)radiotherapy are all counseled by a dietitian during their treatment. Patients undergoing surgery will receive nutritional care as indicated by their MUST score or as indicated by the “Perioperatief voedingsbeleid” guideline: inability to start oral intake within 48 hours after surgery or no more than 50% of the recommended daily energy intake on the fifth day postoperatively [21].

Patients enrolled in the control arm of the study will receive standard care, which means they will not receive any nutritional counseling or nutritional intervention before treatment commences.

5.2 Use of co-intervention (if applicable)

Not applicable.

5.3 Escape medication (if applicable)

Not applicable.

6. INVESTIGATIONAL PRODUCT

Not applicable. The treatment (i.e., nutritional prehabilitation as described in paragraph 5.1) used in this study is part of standard clinical care, applied to patients with head and neck cancer with high risk of malnutrition based on the MUST. The execution of nutritional prehabilitation is in accordance with our current standard clinical practice, in line with local, national and worldwide guidelines.

7. NON-INVESTIGATIONAL PRODUCT

Not applicable. No medicinal products, food products, chemical compounds, stable isotopes or other products are used in an experimental setting. All dietary interventions are administered in compliance with our current standard clinical practice, in line with local, national and worldwide guidelines.

8. METHODS

8.1 Study parameters/endpoints

8.1.1 Main study parameter/endpoint

The primary outcome will be adverse events (intra- and postoperative complications and (chemo)radiotherapy toxicity). Relevant complications include hemorrhage, infection and wound healing disorders including dehiscence and fistula. Complications will be measured within 30 days after surgery using the Clavien-Dindo classification and evaluated during hospital stay and planned and unplanned outpatient visits [22]. Clavien-Dindo grade ≥ 2 complications will be deemed clinically relevant. Toxicity will be evaluated using the Common Terminology Criteria for Adverse Events (CTCAE), version 4.0, at 6 and 12 weeks after the start of (chemo)radiotherapy [23]. The following domains are scored within the CTCAE: xerostomia, taste, throat pain, oral pain, general pain, dysphagia, hoarseness and mucositis. Clinically relevant toxicity will be defined as CTCAE toxicity grade ≥ 3, irrespective of the mentioned domains.

Both complications and (chemo)radiotherapy toxicity are evaluated as part of our current standard practice.

8.1.2 Secondary study parameters/endpoints

Secondary outcome measures will be patient-reported quality of life outcome measures (i.e., the EAT-10, EORTC H&N35 and EORTC C30 questionnaires), recurrence and (disease-specific and overall) survival. Patients will be monitored for two years with intervals of at least three months. These intervals coincide with regular follow-up visits to the outpatient departments. The questionnaires will be fulfilled at baseline and at 3, 6, 12, 18 and 24 months after last treatment. Lastly, the EQ-5D-5L, *i*MCQ and *i*PCQ will be administered to be able to perform a cost-utility and cost-effectiveness evaluation. The EQ-5D-5L, *i*MCQ and *i*PCQ will be fulfilled in the first year of follow-up at baseline and at 3, 6 and 12 months after last treatment. The EORTC H&N35, EORTC C30, EQ-5D-5L questionnaires are part of our current standard practice, as well as the evaluation of recurrence and survival. The EAT-10, *i*MCQ and *i*PCQ are not part of our current standard practice.

8.1.3 Other study parameters

Baseline characteristics include age, gender, smoking status, alcohol use, tumor site, tumor stage, (intended) type of treatment, length, weight (loss), skeletal muscle mass, number of dietetic consultations, dietary intake using 24-hour recall, nutritional status

using the MUST, Global Leadership Initiative on Malnutrition (GLIM)-criteria, Patient Generated Subjective Global Assessment Short Form (PG-SGA SF) score and bio-electrical impedance analyses (BIA)-measurements, physical status using grip strength and Timed Up and Go Test (TUG), and skeletal muscle mass. Dietary intake using 24-hour recall, GLIM-criteria, PG-SGA SF and BIA-measurements will not only be performed at baseline to assess nutritional status, but also at the end of the dietetic intervention (or, for the control group, just before the treatment starts), and three months after the final treatment.

The presence of (radiological) sarcopenia, or more specific skeletal muscle mass, will be measured at baseline and used within sub analysis. In patients with colorectal cancer, sarcopenia tended to be associated with treatment tolerance more often than other screening methods [24]. Sarcopenia is a progressive and generalized loss of muscle function and skeletal muscle mass. In its 2018 sarcopenia definition, the European Working Group on Sarcopenia in Older People 2 (EWGSOP2) requires a decrease in muscle function combined with a decrease in skeletal muscle mass [25]. The Sarcopenia Definitions and Outcomes Consortium, and the Special Interest Group on cachexia-anorexia in chronic wasting diseases of the European Society for Clinical Nutrition and Metabolism (ESPEN) support the use of both grip strength and skeletal muscle mass for defining sarcopenia [26, 27]. In oncology research, a (radiological) sarcopenia diagnosis is usually confirmed by the presence of low muscle quantity, which can be estimated using a variety of techniques, but usually CT is used for non-invasive assessment of muscle quantity. Skeletal muscle mass is most commonly assessed on a single CT slice at the abdominal level of L3, which has been shown to have excellent correlation with whole body skeletal muscle mass as measured using whole body MRI [28]. The cross-sectional skeletal muscle area (CSMA) at the level of L3 is then most commonly normalized for patients' squared height, to calculate the lumbar skeletal muscle index (LSMI). The latter is a surrogate marker for total body skeletal muscle mass [29]. In patients with head and neck cancer, abdominal imaging is not routinely performed, whereas imaging in the head and neck area is routinely performed. Therefore, measurement methods for skeletal muscle mass on a single axial slice at the cervical level of C3 have been developed [30-32]. The CT scans that are used for these measurements are part of our current standard diagnostic work-up. Pre-treatment CSMA at the level of C3 will be determined for each included patient using the methods described in previous papers written by members of our research team [30, 32]. The first slice when scrolling from caudal to cranial direction in the axial plane to show both transverse processes and

the entire vertebral arc will be selected. For CT, muscle tissue will be identified using Hounsfield Unit (HU) range settings from -29 to +150 HU, which is specific for muscle tissue [29]. In a semi-automatic procedure, densities within this range will be delineated. Manual adjustment is necessary to make sure large veins, arteries and lymph nodes are not included within the region of interest. Both sternocleidomastoid muscles and paravertebral muscles will be separately contoured. The CSMA will be calculated as the sum of these structures. The acquired CSMA at the level of C3 will be converted to CSMA at the level of L3 and subsequently corrected for patients' squared height to obtain the LSMI. Low LSMI will be defined as lower than 46.5 cm^2/m^2 for men and lower than 37.9 cm^2/m^2 for women [13]. Typically, 50 to 75% of patients with head and neck cancer have low skeletal muscle mass prior to the start of treatment [33-35]. Low skeletal muscle mass is a phenotypic criterion for malnutrition [36] and is associated with increased rates of intra- and postoperative complications, chemoradiotherapy toxicity and mortality in this patient population [33-35, 37, 38].

Grip strength and BIA-measurements are not part of our current standard practice.

8.2 Randomisation, blinding and treatment allocation

The website randomization tool provided by REDCap will be used. A block randomization will be used with an allocation ratio of 1:1. The randomization chart, including block size, will be established by an independent data manger before the start of the study. Consequently, treatment allocation sequence will be concealed for patients, care providers and researchers (including outcome assessors). The outcome assessors in this study are patients (self-assessment using questionnaires) and care providers (Clavien Dindo classification, CTCAE, recurrence and survival).

8.3 Study procedures

Procedures that are part of standard clinical practice:

- Diagnostic head and neck CT scans, which will be used to measure pre-treatment CSMA at the level of C3 [29, 30, 32]. The acquired CSMA at the level of C3 will be converted to CSMA at the level of L3 and subsequently corrected for patients' squared height to obtain the LSMI. Low LSMI will be defined as lower than 46.5 cm^2/m^2 for men and lower than 37.9 cm^2/m^2 for women [13];
- Intra- and postoperative complications measured within 30 days after surgery using the Clavien-Dindo classification;

- (Chemo)radiotherapy toxicity measured 6 and 12 weeks after the start of (chemo)radiotherapy using the CTCAE version 4.0;
- EORTC H&N35 measured for two years at baseline and at 3, 6, 12, 18 and 24 months;
- EORTC C30 measured for two years at baseline and at 3, 6, 12, 18 and 24 months;
- EQ-5D-5L measured at baseline and at 3, 6, 12, 18 and 24 months.

Procedures that are not part of standard clinical practice and all of which will not postpone diagnostic procedures or treatment:

- Grip strength, which will be measured using a Jamar hydraulic handheld dynamometer as recommended by the American Society of Hand Therapists and expressed in kilograms. Patients will be asked to squeeze maximally with each hand. Each hand will be measured three times. Low grip strength will be defined as lower than 27 kg in men and lower than 16 kg in women [25]. This measurement will be performed at baseline and takes less than five minutes to complete;
- BIA-measurements to estimate body composition, including body fat and muscle mass, using a weak electric current that flows through the body. The measurement will be performed at baseline, just before the treatment starts, and three months after the final treatment. It takes on average 15 minutes to complete;
- EAT-10 in the first two years of follow-up at baseline and at 3, 6, 12, 18 and 24 months after last treatment. The EAT-10 consists of 10 questions in three domains: functional, emotional and physical. Time to complete the EAT-10 is on average two minutes;
- *i*MCQ in the first year of follow-up at baseline and at 3, 6 and 12 months after last treatment. The *i*MCQ consists of 20 questions. Time to complete the *i*MCQ is on average 10 minutes;
- *i*PCQ in the first year of follow-up at baseline and at 3, 6 and 12 months after last treatment. The *i*PCQ consists of 18 questions, of which nine are general questions to collect demographic information, such as age, sex and work status. Productivity losses are measured in three separate modules (absenteeism, presenteeism and productivity losses of unpaid work). Time to complete the *i*PCQ is on average 5 minutes.

8.4 Withdrawal of individual subjects

Subjects can leave the study at any time for any reason if they wish to do so without any consequences. The investigator can decide to withdraw a subject from the study for urgent medical reasons.

8.4.1 Specific criteria for withdrawal (if applicable)

Not applicable.

8.5 Replacement of individual subjects after withdrawal

To anticipate withdrawal of 5% of subjects, three more subjects than needed will be recruited per group. In case of withdrawal before randomization, the subject will be replaced for a new subject. If more than 5% of subjects withdraw before completing the intervention (i.e., nutritional prehabilitation) or before completing the primary endpoint (intra- and postoperative complications within 30 days after surgery or toxicity six weeks after the start of (chemo)radiotherapy), they will be replaced with additional subjects to ensure a total of 128 subjects for the evaluation of the primary endpoint.

8.6 Follow-up of subjects withdrawn from treatment

Subjects withdrawn from treatment will continue to receive the standard medical treatment and five-year follow-up provided by their head and neck surgeon and/or radiotherapist and/or medical oncologist. They will no longer have to complete the questionnaires that are part of this study.

8.7 Premature termination of the study

Reasons for premature termination of the study may be:

- If the positive decision of the METC is irrevocably revoked;
- If a reasonable case can be made for terminating the study in the interests of the health of the subjects:
    - o If continuation of the study does not serve any scientific purpose and this is confirmed by the METC that has issued a positive decision on the study;
    - o If the subsidising party has been declared insolvent;
    - o If either the sponsor or the subsidising party is dissolved as a legal entity;

- If the project leader is no longer capable of performing the tasks of project leader and no replacement agreeable to all parties can be found;
- If circumstances beyond the control of the sponsor, investigator or subsidising party make it unreasonable to continue the study.

9. SAFETY REPORTING

9.1 Temporary halt for reasons of subject safety

In accordance with section 10, subsection 4, of the WMO, the sponsor will suspend the study if there is sufficient ground that continuation of the study will jeopardise subject health or safety. The sponsor will notify the accredited METC without undue delay of a temporary halt including the reason for such an action. The study will be suspended pending a further positive decision by the accredited METC. The investigator will take care that all subjects are kept informed.

9.2 AEs, SAEs and SUSARs

9.2.1 Adverse events (AEs)

Adverse events are defined as any undesirable experience occurring to a subject during the study, whether or not considered related to the experimental intervention (i.e., nutritional prehabilitation). All adverse events reported spontaneously by the subject or observed by the investigator or his staff will be recorded up until 6 weeks after last treatment, thereafter only SAEs will be recorded.

Any event involving adverse drug reactions, illnesses with onset during the study or exacerbations of pre-existing illnesses will be recorded, including but not limited to clinically significant changes in physical examination findings and abnormal objective test findings (e.g., x-ray, ECG). The criteria for determining whether an abnormal objective test finding should be reported as an AE are as follows:

- The test result is associated with clinically significant symptoms, and/or;
- The test result leads to a discontinuation from the clinical trial, significant additional concomitant drug treatment or other therapy and/or;
- The test result leads to any of the outcomes included in the definition of a SAE, and/or;
- The test result is considered to be clinically relevant by the investigator.

9.2.2 Serious adverse events (SAEs)

A serious adverse event is any untoward medical occurrence or effect that:

- results in death;
- is life threatening (at the time of the event);
- requires hospitalisation or prolongation of existing inpatients' hospitalisation;
- results in persistent or significant disability or incapacity;

- is a congenital anomaly or birth defect; or
- any other important medical event that did not result in any of the outcomes listed above due to medical or surgical intervention but could have been based upon appropriate judgement by the investigator.

An elective hospital admission will not be considered as a serious adverse event.

The investigator will report all SAEs to the sponsor without undue delay after obtaining knowledge of the events, except for the following SAEs:

- (prolonged) hospitalisation or intervention due to: postoperative hemorrhage, infection and wound healing disorders, which all can be expected with standard of care (i.e., (major) head and/or neck surgery).
- hospitalisation or intervention due to: (chemo)radiotherapy toxicity such as vomiting, throat pain, oral pain, dysphagia, mucositis and blood value imbalances, which all can be expected with standard of care (i.e., 70 Gy (chemo)radiotherapy).

The sponsor will report the SAEs through the web portal *ToetsingOnline* to the accredited METC that approved the protocol, within 7 days of first knowledge for SAEs that result in death or are life threatening followed by a period of maximum of 8 days to complete the initial preliminary report. All other SAEs will be reported within a period of maximum 15 days after the sponsor has first knowledge of the serious adverse events.

9.2.3 Suspected unexpected serious adverse reactions (SUSARs)

Not applicable.

9.3 Annual safety report

Not applicable.

9.4 Follow-up of adverse events

All AEs will be followed until they have abated, or until a stable situation has been reached. Depending on the event, follow up may require additional tests or medical procedures as indicated, and/or referral to the general physician or a medical specialist. SAEs need to be reported till end of study within the Netherlands, as defined in the protocol.

9.5 Data Safety Monitoring Board (DSMB) / Safety Committee

A DSMB is not needed. This study is considered a negligible-risk study: there is a small chance of damage and damage is expected to slight. Therefore, an internal monitor will be used who has completed the basic training for monitors in the UMCG. Monitoring will be performed by an independent monitor.

10. STATISTICAL ANALYSIS

Baseline characteristics, primary outcomes and secondary outcomes are quantitative and will be presented categorical and continuous. Categorical variables will be presented as absolute numbers and percentages of the total. Continuous variables will be tested for normality using Kolmogorov-Smirnov analysis. Continuous variables will be presented as means and standard deviations for normally distributed data, and medians and interquartile ranges for data that is not normally distributed. Between-group mean (or median) differences, rate differences and rate ratios with 95% confidence intervals (or ranges) will be calculated.

Missing values will be handled using multiple imputation, assuming that there is only missingness at random, and all analyses will be performed on an intention-to-treat basis. The missingness at random assumption will be tested by exploring patterns of missing data using missingness patterns plots and missingness indicators, as well as performing the Little's MCAR test. If the missingness at random assumption had been violated, we will perform Heckman regression.

Intercurrent events may affect the estimated treatment effect. An expected intercurrent event is non-adherence to the study intervention or the use of certain foods or medications outside the advice given by the dieticians. In the case of non-adherence to the study intervention or the use of certain foods or medications, a 'treatment policy' strategy will be used in which we accept that this intercurrent event is part of the intervention and we expect that non-adherence to the intervention also occurs in daily practice. Loss of follow-up because participants decide to stop the study is in our opinion not an intercurrent event but a 'missing data' problem.

SPSS version 28.0 (SPSS Inc., Chicago, IL, USA) will be used for all statistical analyses.

10.1 Primary study parameter(s)

The primary outcome will be adverse events (intra- and postoperative complications and (chemo)radiotherapy toxicity). Relevant complications include hemorrhage, infection and wound healing disorders including dehiscence and fistula. Complications will be measured within 30 days after surgery using the Clavien-Dindo classification and evaluated during hospital stay and planned and unplanned outpatient visits [22]. Clavien-Dindo grade ≥ 2 complications will be deemed clinically relevant. Toxicity will be evaluated using the Common Terminology Criteria for Adverse Events (CTCAE), version

4.0, 6 and 12 weeks after the start of (chemo)radiotherapy. The following domains are scored within the CTCAE: xerostomia, taste, throat pain, oral pain, general pain, dysphagia, hoarseness and mucositis. Clinically relevant toxicity will be defined as CTCAE toxicity grade ≥ 3, irrespective of the mentioned domains.

For analysis of between-group differences in the primary outcome, a logistic regression analysis will be performed. The endpoints complications and toxicity will be dichotomized. In case of unbalance between the intervention and the control group, adjusted logistic regression analyses will be performed. A *p*-value < 0.05 will be considered as statistically significant.

Subgroup analyses will be performed using the following variables:

- (intended) type of treatment (surgery versus (chemo)therapy
- Subsite (larynx versus hypopharynx versus oropharynx versus oral cavity)
- Skeletal muscle mass (LSMI in cm2/m2)
- Duration of nutritional prehabilitation intervention
- Number of dietetic consultations
- Dietary intake using 24-hour recall

These variables will be added to the regression analyses

10.2 Secondary study parameter(s)

Secondary outcome measures will be patient-reported quality of life outcome measures (i.e., the EAT-10, EORTC H&N35 and EORTC C30 questionnaires), recurrence and (disease-specific and overall) survival. Patients will be monitored for two years with intervals of at least three months. For further analyses of between-group differences in these secondary outcomes, logistic regression analyses will be performed for categorical outcomes, cox proportional hazard regression for time-dependent outcomes (i.e., recurrence and survival) and linear regression analyses for continuous variables. A *p*-value < 0.05 will be considered as statistically significant.

To correct for false discovery rate, the Benjamini-Hochberg procedure will be applied.

Furthermore, an economic evaluation will be performed to compare the incremental cost-effectiveness of nutritional prehabilitation as compared to standard care. A cost-utility analysis (CUA) and cost-effectiveness analysis (CEA) will be conducted from a societal perspective. The CUA will be performed based on EQ-5D-5L defined utilities over a time

horizon of one year. The CEA will incorporate the highest measured grade of complications (Clavien-Dindo classification) at 30 days postoperatively or (chemo)radiotherapy toxicities (CTCAE) at 6 and 12 weeks after the start of (chemo)radiotherapy. Health care consumption will be measured on a patient level and registered on a case-report form and partly on questionnaires (*i*MCQ items). Productivity losses will be measured using the *i*PCQ. Costs will be calculated according to the Dutch guidelines for costing research in health economic evaluations, issued by the National Healthcare Institute [39]. There will be no discounting since the time horizon of the analyses does not exceed one year. Cost-effectiveness planes and acceptability curves will be plotted and 95% confidence intervals will be based on bootstrapping.

10.3 Interim analysis (if applicable)

Not applicable.

11. ETHICAL CONSIDERATIONS

11.1 Regulation statement

The study will be conducted according to the principles of the Declaration of Helsinki (amended at the 64th WMA General Assembly, Fortaleza, Brazil, October 2013) and in accordance with the principles of ‘Good Clinical Practice’ and the Medical Research Involving Human Subjects Act (WMO).

11.2 Recruitment and consent

Potentially eligible patients will be identified by their treating physician. Eligible patients will be informed about the study by their treating physician, after which they will receive the patient information folder and two copies of the informed consent form from their treating physician.

Patients will be given one week to consider their decision. Further information will be provided and questions will be answered where necessary by the treating physician or independent expert, upon the patient’s initiative. If the patient wishes to participate, they will be asked to hand over a signed informed consent form during their next visit to the outpatient department or send a signed informed consent form using the provided return envelope. The informed consent form will then be signed by the investigator upon receiving it.

Definitive eligibility is assessed by one of the investigators in consultation with the treating physician using the in- and exclusion criteria stated in this protocol after informed consent has been obtained.

11.3 Objection by minors or incapacitated subjects (if applicable)

Not applicable.

11.4 Benefits and risks assessment, group relatedness

We expect that nutritional prehabilitation will lead to fewer (serious) complications and side effects compared to no nutritional prehabilitation. We expect a higher quality of life, equal chance of recurrence and better survival.

11.4.1 Risk assessment

There is extensive experience with the dietary measures as outlined in 5.1. They are based on local, national and worldwide protocols and guidelines on nutritional support for (head and neck) cancer patients. All patients with high risk of malnutrition based on the

MUST receive dietary measures. Patients with high risk of malnutrition are frailer and more frequently experience swallowing problems than the study population. The study population is a low-risk patient group compared to the patient group already receiving the intervention.
Oral nutritional supplements (e.g., nutridrinks) and enteral nutritional support used in this study comply with the FDA regulations regarding ingredients, labelling and manufacturing process.
Common potential adverse events/risks of the use of oral nutritional supplements are excessive mucus formation. The use of oral nutritional supplements is not expected to increase swallowing difficulties. Common potential adverse events/risk of the use of enteral nutritional support are nausea, vomiting, diarrhoea, constipation, irritation of the nose of throat, a blocked feeding tube needing replacement. Adverse events will be monitored during consultations with the dietitian and medical specialists.

Given the extensive experience with the intervention in a patient population that is high-risk compared to the study population and the small change of adverse events that cause slight damage at most, we think this study can be considered a negligible-risk study.

11.5 Compensation for injury

The sponsor/investigator has a liability insurance which is in accordance with article 7 of the WMO.

The sponsor (also) has an insurance which is in accordance with the legal requirements in the Netherlands (Article 7 WMO). This insurance provides cover for damage to research subjects through injury or death caused by the study.
The insurance applies to the damage that becomes apparent during the study or within 4 years after the end of the study.

11.6 Incentives (if applicable)

Not applicable.

12. ADMINISTRATIVE ASPECTS, MONITORING AND PUBLICATION

12.1 Handling and storage of data and documents

All data will be handled confidentially and a patient identification code list will be used to link the data to the subject. The code will not be based on the patient's initials or birthdate. The key to the code will be safeguarded by the project leader and will be saved on a research drive at the UMCG. The data will be coded using numbers in order of enrolment. Only the investigators mentioned in this proposal and the monitor will have access to the source data. Handling of personal data will comply with the EU General Data Protection Regulation and the Dutch Act on Implementation of the General Data Protection Regulation (in Dutch: Uitvoeringswet AVG, UAVG). Data will be stored for 15 years in a locked office at the UMCG, on a research drive especially made for research data storage.

12.2 Monitoring and Quality Assurance

This study can be classified as a low-risk study and therefore monitor visits will be performed by an internal monitor at the UMCG appointed through the Central Research Office. Details will be provided through the monitoring plan.

12.3 Amendments

Amendments are changes made to the research after a favourable opinion by the accredited METC has been given. All amendments will be notified to the METC that gave a favourable opinion.

12.4 Annual progress report

The sponsor/investigator will submit a summary of the progress of the trial to the accredited METC once a year. Information will be provided on the date of inclusion of the first subject, numbers of subjects included and numbers of subjects that have completed the trial, serious adverse events/ serious adverse reactions, other problems, and amendments.

12.5 Temporary halt and (prematurely) end of study report

The investigator/sponsor will notify the accredited METC of the end of the study within a period of 8 weeks. The end of the study is defined as the last patient's last visit.
The sponsor will notify the METC immediately of a temporary halt of the study, including the reason of such an action.
In case the study is ended prematurely, the sponsor will notify the accredited METC within 15 days, including the reasons for the premature termination.

Within one year after the end of the study, the investigator/sponsor will submit a final study report with the results of the study, including any publications/abstracts of the study, to the accredited METC.

12.6 Public disclosure and publication policy

The knowledge or skills that will be obtained during this project will not be protected through patents or otherwise. The project leader together with other members of the research team involved will review the data and perform the statistical analyses. Manuscripts will be submitted to open-access, peer-reviewed journals, regardless of the results. There will be no restrictions towards publication. Results will be published by the principal investigators and PhD student appointed to the project, plus additional research team members that contributed substantially to the study design, analyses, interpretation of study results and writing process.

13. REFERENCES

1. Bray, F., Ferlay, J., Soerjomataram, I., Siegel, R.L., Torre, L.A., Jemal, A. (2018) Global cancer statistics 2018: GLOBOCAN estimates of incidence and mortality worldwide for 36 cancers in 185 countries. *CA Cancer J. Clin*. 68, 394-424.

2. Jager-Wittenaar, H., Dijkstra, P.U., Vissink, A., van Oort, R.P., van der Laan, B.F.A.M., Roodenburg, J.L.N. (2011) Malnutrition in patients treated for oral or oropharyngeal cancer--prevalence and relationship with oral symptoms: an explorative study. *Support Care Cancer* 19, 1675-1683.

3. Jager-Wittenaar, H., Dijkstra, P.U., Vissink, A., Langendijk, J.A., van der Laan, B.F.A.M., Pruim, J., Roodenburg, J.L.N. (2011) Changes in nutritional status and dietary intake during and after head and neck cancer treatment. *Head Neck* 33, 863-870.

4. Alshadwi, A., Nadershah, M., Carlson, E.R., Young, L.S., Burke, P.A., Daley, B.J. (2013) Nutritional considerations for head and neck cancer patients: a review of the literature. *J. Oral Maxillofac. Surg*. 71, 1853-1860.

5. Hébuterne, X., Lemarié, E., Michallet, M., Beauvillain de Montreuil, C., Schneider, S.M., Goldwasser, F. (2014) Prevalence of malnutrition and current use of nutrition support in patients with cancer. *J. Parenter. Enteral Nutr*. 38, 196-204.

6. Jager-Wittenaar, H., Dijkstra, P.U., Vissink, A., van der Laan, B.F.A.M., van Oort, R.P., Roodenburg, J.L.N. (2011) Malnutrition and quality of life in patients treated for oral or oropharyngeal cancer. *Head Neck* 33, 490-496.

7. Langius, J.A.E., Bakker, S., Rietveld, D.H.F., Kruizenga, H.M., Langendijk, J.A., Weijs, P.J.M., Leemans C.R. (2013) Critical weight loss is a major prognostic indicator for disease-specific survival in patients with head and neck cancer receiving radiotherapy. *Br. J. Cancer* 109, 1093-1099.

8. Kono, T., Sakamoto, K., Shinden, S., Ogawa, K. (2017) Pre-therapeutic nutritional assessment for predicting severe adverse events in patients with head and neck cancer treated by radiotherapy. *Clin. Nutr*. 36, 1681-1685.

9. Landelijke Werkgroep Diëtisten Oncologie. (2012) Richtlijn “Ondervoeding bij kanker”. https://richtlijnendatabase.nl/richtlijn/ondervoeding_bij_patienten_met_kanker/ondervoeding_-_korte_beschrijving.html.

10. Bras, L., de Vries, J., Festen, S., Steenbakkers, R.J.H.M., Langendijk, J.A., Witjes, M.J.H., van der Laan, B.F.A.M., de Bock, G.H., Halmos, G.B. (2021) Frailty and restrictions in geriatric domains are associated with surgical complications but not with radiation-induced acute toxicity in head and neck cancer patients: a prospective study. *Oral Oncol.* 118, 105329.

11. Lambert, J.E., Hayes L.D., Keegan, T.J., Subar, D.A., Gaffney, C.J. (2021) The impact of prehabilitation on patient outcomes in hepatobiliary, colorectal and upper gastrointestinal cancer surgery: a PRISMA-accordant meta-analysis. *Ann. Surg.* 274, 70-77.

12. Dewulf, M., Verrips, M., Coolsen, M.M.E., Olde Damink, S.W.M., den Dulk, M., Bongers, B.C., Dejong, K., Bouwense, S.A.W. (2021) The effect of prehabilitation on postoperative complications and postoperative hospital stay in hepatopancreatobiliary surgery a systematic review. *HPB (Oxford)* 23, 1299-1310.

13. Zwart, A.T., Portzgen, W., van Rijn-Dekker, I., Sidorenkov, G.A., Dierckx, R.A.J.O., Steenbakkers, R.J.H.M., Wegner, I., van der Hoorn, A., de Bock, G.H., Halmos, G.B. (2022) Determining gender-specific cut-off values for low skeletal muscle mass to identify patients at risk for treatment-related adverse events in head and neck cancer. *J. Clin. Medicine* 11, 4650.

14. Surov, A., Wienke, A. (2021) Low skeletal muscle mass predicts relevant clinical outcomes in head and neck squamous cell carcinoma. A meta analysis. *Ther. Adv. Med. Oncol.* 13, 17588359211008844.

15. FAO/WHO/UNU. (1985) Energy and protein requirements. Report of a joint FAO/WHO/UNU Expert Consultation. *World Health Organ. Tech. Rep. Ser.* 724, 1–206.

16. Muscaritoli, M., Arends, J., Bachmann, P., et al. (2021) ESPEN practical guideline: Clinical Nutrition in cancer. *Clin. Nutr.* 40, 2898-2913.

17. National Institute for Health and Clinical Excellence. (2017) Nutrition support for adults: oral nutrition support, enteral tube feeding and parenteral nutrition. https://www.nice.org.uk/guidance/cg32.

18. Dekker, I.M., van Rijssen, N.M., Verreijen, A., Weijs, P.J., de Boer, W.B.E., Terpstra, D., Kruizenga, H.M. (2022) Calculation of protein requirements; a comparison of calculations based on bodyweight and fat free mass. Clin. Nutr. ESPEN. 48, 378-385.

19. Gallagher, D., Heymsfield, S.B., Heo, M., Jebb, S.A., Murgatroyd, P.R., Sakamoto, Y. (2000) Healthy percentage body fat ranges: an approach for developing guidelines based on body mass index. *Am. J. Clin. Nutr.* 72, 694-701.

20. Nederlands Voedingsteam Overleg. (2019) NVO Richtlijn Refeedingsyndroom. https://nederlandsvoedingsteamoverleg.nl/nvo-richtlijnen/refeeding-syndroom.

21. Nederlandse Vereniging voor Anesthesiologie. (2022) Richtlijn perioperatief voedingsbeleid. https://richtlijnendatabase.nl/richtlijn/perioperatief_voedingsbeleid/deel_ii_-_pre-_en_postoperatief_voedingsbeleid_bij_volwassenen_exclusief_ic/optimaliseren_postoperatief_voedingsbeleid_bij_chirurgische_patienten.html.

22. Monteiro, E., Sklar, M.C., Eskander, A., de Almeida, J.R., Shrime, M., Gullane, P., Irish, J., Gilbert, R., Brown, D., Higgins, K., Enepekides, D., Goldstein, D.P. (2014) Assessment of the Clavien-Dindo classification system for complications in head and neck surgery. *Laryngoscope* 124, 2726–1231.

23. National Cancer Institute. (2009) Common Terminology Criteria for Adverse Events (CTCAE) version 4.0. https:// evs.nci.nih.gov/ftp1/CTCAE/CTCAE_4.03/Archive/CTCAE_4.0_2009-05-29_ QuickReference_8.5x11.pdf.

24. Beukers, K., Voorn, M.J.J., Trepels, R., van de Wouw, A.J., Vogelaar, F.J., Havermans, R.C., Janssen-Heijnen, M.L.G. (2022) Associations between outcome variables of nutritional screening methods and systematic treatment tolerance in patients with colorectal cancer: a systematic review. *J. Geriatr. Oncol.* S1879-4068(22)00147-3.

25. Cruz-Jentoft, A.J., Bahat, G., Bauer, J., et al; writing group for the European Working Group on Sarcopenia in Older People 2 (EWGSOP2), and the extended group for EWGSOP2. (2019) Sarcopenia: revised European consensus on definition and diagnosis. *Age Ageing* 48, 601.

26. Bhasin, S., Travison, T.G., Manini, T.M., et al. (2020) Sarcopenia definition: the position statements of the Sarcopenia Definition and Outcomes Consortium. *J. Am. Geriatr. Soc*. 68, 1410–1418.

27. Muscaritoli, M., Anker, S.D., Argilés, J., et al. (2010) Consensus definition of sarcopenia, cachexia and pre-cachexia: joint document elaborated by Special Interest Groups (SIG) “cachexia-anorexia in chronic wasting diseases” and “nutrition in geriatrics”. *Clin. Nutr*. 29, 154–159.

28. Prado, C.M., Lieffers, J.R., McCargar, L.J., Reiman, T., Sawyer, M.B., Martin, L., Baracos, V.E. (2008) Prevalence and clinical implications of sarcopenic obesity in patients with solid tumours of the respiratory and gastrointestinal tracts: a population-based study. *Lancet Oncol*. 9, 629-635.

29. Mitsiopoulos, N., Baumgartner, R.N., Heymsfield, S.B., Lyons, W., Gallagher, D., Ross, R. (1998) Cadaver validation of skeletal muscle measurement by magnetic resonance imaging and computerized tomography. *J. Appl. Physiol.* 85, 115-122.

30. Swartz, J.E., Pothen, A.J., Wegner, I., Smid, E.J., Swart, K.M.A., de Bree, R., Leenen, L.P.H., Grolman, W. (2016) Feasibility of using head and neck CT imaging to assess skeletal muscle mass in head and neck cancer patients. *Oral Oncol*. 62, 28-33.

31. Bril, S.I., Chargi, N., Wendrich, A.W., Wegner, I., Bol, G.H., Smid, E.J., de Jong, P.A., Devriese, L.A., de Bree, R. (2021) Validation of skeletal muscle mass assessment at the level of the third cervical vertebra in patients with head and neck cancer. *Oral Oncol.* 123, 105617.

32. Zwart, A.T., Becker, J.N., Lamers, M.J., Dierckx, R.A.J.O., de Bock, G.H., Halmos, G.B., van der Hoorn, A. (2021) Skeletal muscle mass and sarcopenia can be determined with 1.5-T and 3-T neck MRI scans, in the event that no neck CT scan is performed. *Eur. Radiol*. 31, 4053-4062.

33. Wendrich, A.W., Swartz, J.E., Bril, S.I., Wegner, I., de Graeff, A., Smid, E.J., de Bree, R., Pothen, A.J. (2017) Low skeletal muscle mass is a predictive factor for chemotherapy dose-limiting toxicity in patients with locally advanced head and neck cancer. *Oral Oncol*. 71, 26-33.

34. Bril, S.I., Pezier, T.F., Tijink, B.M., Janssen, L.M., Braunius, W.W., de Bree, R. (2019) Preoperative low skeletal muscle mass as a risk factor for pharyngocutaneous fistula and decreased overall survival in patients undergoing total laryngectomy. *Head Neck* 41, 1745-1755.

35. Chargi, N., Bril, S.I., Swartz, J.E., Wegner, I., Willems, S.M., de Bree, R. (2020) Skeletal muscle mass is an imaging biomarker for decreased survival in patients with oropharyngeal squamous cell carcinoma. *Oral Oncol*. 101, 104519.

36. Cederholm, T., Jensen, G.L., Correia, M.I.T.D., et al; GLIM Core Leadership Committee; GLIM Working Group. (2019) GLIM criteria for the diagnosis of malnutrition – A consensus report from the global clinical nutrition community. *Clin. Nutr*. 38, 1-9.

37. Huiskamp, L.F.J., Chargi, N., Devriese, L.A., de Jong, P.A., de Bree, R. (2020) The predictive and prognostic value of low skeletal muscle mass for dose-limiting toxicity and survival in head and neck cancer patients receiving concomitant cetuximab and radiotherapy. Eur. Arch. Otorhinolaryngol. 277, 2847-2858.

38. Sealy, M.J., Dechaphunkul, T., van der Schans, C.P., Krijnen, W.P., Roodenburg, J.L.N., Walker, J., Jager-Wittenaar, H., Baracos, V.E. (2020) Low muscle mass is associated with early termination of chemotherapy related to toxicity in patients with head and neck cancer. Clin. Nutr. 39, 501-509.

39. Hakkaart-van Roijen, L., van der Linden, N., Bouwmans, C., Kanters, T., Tan, S.S. (2016) Kostenhandleiding: methodologie van kostenonderzoek en referentieprijzen voor economische evaluaties in de gezondheidszorg. https://www.zorginstituutnederland.nl/binaries/zinl/documenten/publicatie/2016/02/29/richtlijn-voor-het-uitvoeren-van-economische-evaluaties-in-de-gezondheidszorg/Richtlijn%2Bvoor%2Bhet%2Buitvoeren%2Bvan%2Beconomische%2Bevaluaties%2Bin%2Bde%2Bgezondheidszorg%2B%28verdiepingsmodules%29.pdf.
